# Supplementary material for: Cell salvage in bacterially contaminated surgical fields – A scoping review
Source: PLoS One. 2026 Jan 5;21(1):e0339574. doi: 10.1371/journal.pone.0339574 (PMC12768276; doi:10.1371/journal.pone.0339574)
Supplement: S1 Table — (DOCX) [file pone.0339574.s001.docx]

**Supplemental Table 1**

*Bacterial species identified in final product.*

| First author | Year | Bacteria identified in final product | |  |
| --- | --- | --- | --- | --- |
| CARDIOVASCULAR SURGERY | | |  | |
| Khan | 1975 | Diphteroids, S. albus (no numbers stated) | | |
| Davies | 1987 | S. epidermidis (7), Corynebacterium (1), Flavobacterium (1), Propionibacterium (1), S. faecalis/P. mirabilis (1), Listeria monocytogenes/propionobacterium (1) | | |
| Ezzedine | 1991 | CNS (41), Micrococcus spp. (5), Diphtheroids (3), A. lwoffii (1), Bacillus spp. (1) | | |
| Bland | 1992 | S. epidermidis (13), CNS (8), P. acnes (4), Diphtheroids (3), S. capitis (3), S. hominis (2), , S. warneri (2), S. haemolyticus (2), E. agglomerans (1), P. aeruginosa (1), S. saccharolyticus (1), S. simulans (1) | | |
| Reents | 1999 | S. epidermidis, Propionibacteria species, Corynebacterium species, Peptococcus species (no numbers stated) | | |
| Shindo | 2004 | S. epidermidis (30), Bacillus species (3), Micrococcus (1), Bacteroides (1), anaerobic Gram+ rods (1) | | |
| Ishida | 2006 | NR | | |
| Schmidt | 2009 | CNS (1), Propionibacteria (1) | | |
| Luque-Oliveros | 2020 | S. epidermidis (138), S. sanguinis (S. viridans) (20) | | |
| Zhou | 2023 | S. epidermidis (64), S. haemolyticus (6), S. caprae (3), S. hominis (8), S. cohnii (2), S. capitis (5), S. saccharolyticus (4), M. luteus (3), Str. viridans (2), C. diphtheriae (1), B. fragilis (1), A. ursingii (1) | | |
| LIVER SURGERY | | |  | |
| Kang | 1991 | Phase 1: S. epidermidis in all positive cultures  Phase 2: S. epidermidis (3) | | |
| Feltracco | 2007 | S. epidermidis (10), S. hominis (5), S. haemolyticus (3), S. saprophyticus (1), S. warneri (1), E. coli (1), P. acnes (1), C. albicans (2), C. albicans + S. haemolyticus (2) | | |
| Schmidt | 2009 | CNS (2), MC (1) | | |
| Liang | 2010 | E. coli (1), K. pneumoniae (1), and S. maltophilia (1) | | |
| Kim | 2022 | None | | |
| Kim | 2024 | S. epidermidis (2), E. faecium (2), E. coli (1), S. mitis/oralis (1), Sten. maltophilia (1), S. capitis (1), A. baumannii (1) | | |
| ORTHOPAEDIC SURGERY | | |  | |
| Wollinsky | 1997 | NR | | |
| Nosanchuk | 2001 | Diphtheroids (16), diphteroids + micrococci (1), CNS (1), diptheroids + coagulase-neg. staphylococci (1) | | |
| Perez-Ferrer | 2016 | CNS (12), CNS + enterococcus (1) | | |
| Perez-Ferrer | 2017 | CNS (5) | | |
| Kruger | 2024 | In n=14 patients: S. epidermidis (6), S. capitis (2), C.. acnes (2), c. albicans (2), S. hominis (1), S. aureus (1), S. haemolyticus (1), Gram-positive bacilli (1) | | |
| TRAUMA SURGERY | | |  | |
| Timberlake | 1988 | S. epidermidis, S. aureus, E. coli, Enterococcus, Clostridia, Bacteroides, Streptococcus, Candida, Enterobacter (no numbers stated) | | |
| Ozmen | 1992 | NR | | |
| Bowley | 2006 | CNS (5), yeast (2), E. coli (1), E. coli + M. morganni + E. faecium + yeast (1), E. coli + A. faecalis + Clostridium spp. (1), E. coli + E. faecium + Klebsiella (1) | | |
| GYNAECOLOGY | | |  | |
| Yamada | 1997 | No-antibiotics in n=11 patients: Staphylococcus sp. (7); A. putrefaciens (2); P. aeruginosa (1); Bacillus sp. (1); Gram(+) bacillus (1); P. fluorescens (1);  With antibiotics in n=22 patients: Staphylococcus sp. (7); Gram(+) bacillus (3); Enterococcus sp. (1); P. aeruginosa (1); S. aureus (1) | | |
| Waters | 2000 | Staphylococcus spp. (7) | | |
| Teare | 2015 | In n=50 patients: E. coli (39), Enterococcus spp. (36), CNS (11), Lactobacillus spp. (3), Mixed anaerobes (2), Coliforms (1), Alpha haemolytic streptococci (1), Group B streptococci (1), Proteus (1) | | |
| EAR, NOSE AND THROAT SURGERY & ORAL AND MAXILLOFACIAL SURGERY | | | | |
| Locher | 1992 | In n=18 patients: S. viridans (15), CNS (14), Neisseria (12), S. aureus (6), Haemophilus (5), Corynebacterium (5) | | |
| Lenzen | 2006 | NA | | |
| Wasl | 2016 | S. aureus (2) | | |
| MIXED/OTHER | | |  | |
| Jeng | 1998 | Gram–bacilli + Gram+cocci (1); S. aureus + E. coli (1); S. aureus + nonhemolytic Streptococcus (1); E. coli + P. mirabilis + CNS + S. viridans (1); S. aureus + S. epidermidis (1); K. pneumonia (1); Enterococcus + CNS (1); E. coli, S. viridans, Streptococcus group B (1) | | |
| Sugai | 2001 | S. epidermidis (8), other gram + bacteria (1), other (1) | | |
| Kudo | 2004 | S. epidermidis (9), S. epidermidis + S. viridans (2), S. caceolyticus (1), S. aureus + S. viridans + E. cloacae (1), S. aureus (1) | | |
| Gigengack | 2021 | S. aureus (15), E. faecalis (9), S. epidermidis (9), C. koserii (5), M. morganii (3), P. aeruginosa (3), S. mitis/oralis (3), A. baumannii (2), B. cereus group (2), E. coli (2), H. streptococcus G (2), C. utilis (1), C. striatum (1), E. cloacae complex (1), E. casseliflavus (1), E. faecium (1), E. gallinarum (1), G. adiacens (1), K. pneumoniae (1), P. mirabilis (1), S. caprae (1), S. haemolyticus (1), S. maltophilia (1), S. gallolyticus (1). | | |
