# Supplementary material for: Cell salvage in bacterially contaminated surgical fields – A scoping review
Source: PLoS One. 2026 Jan 5;21(1):e0339574. doi: 10.1371/journal.pone.0339574 (PMC12768276; doi:10.1371/journal.pone.0339574)
Supplement: S2 Table — (DOCX) [file pone.0339574.s002.docx]

**Supplemental Table 2**

*Contamination rates pre- and post-wash*

| First author | Year | Pre-wash contamination | Post-wash contamination | Final contamination | Notes |
| --- | --- | --- | --- | --- | --- |
| CARDIOVASCULAR SURGERY | | |  |  |  |
| Bland | 1992 | NR | 30/31 (96.8) | 30/31 (96.8) | 4/37 of preoperative blood samples culture positive |
| Reents | 1999 | 4/10 (40) | 9/10 (90) | 9/10 (90) |  |
| Schmidt | 2009 | 1/6 (16.7) | 2/6 (33.3) | 2/6 (33.3) |  |
| LIVER SURGERY | | |  |  |  |
| Kang | 1991 | Phase 1:  Aerobic: 4/14 (28.6) Anaerobic: 5/14 (35.7)  Phase 2:  0/11 (0) | Phase 1:  Aerobic: 6/14 (42.9)  Anaerobic: 2/14 (14.2)  Phase 2: 3/11 (27.2) | Phase 1:  Aerobic: 6/14 (42.9)  Anaerobic: 2/14 (14.2) Phase 2: 3/11 (27.2) |  |
| Feltracco | 2007 | NR | 26/38 (68.4) | 26/38 (68.4) | 2/38 CVC samples at beginning of procedure positive |
| Schmidt | 2009 | 3/6 (50) | 3/6 (50) | 3/6 (50) |  |
| Liang | 2010 | 28/45 (62.2) | 15/45 (33.3) | 3/45 (6.7) |  |
| Kim | 2022 | NR | 13/29 (44.8) before BDA; 9/29 (31.0) after BDA | 0/29 (0) before BDA; 0/29 (0) after BDA |  |
| Kim | 2024 | NR | 7/30 (23.3) before graft reperfusion; 11/30 (36.7) after graft reperfusion (P = 0.25) | 7/30 (23.3) (conversion 36.4%, P = 0.25) |  |
| ORTHOPAEDIC SURGERY | | |  |  |  |
| Wollinsky* | 1997 | SG: 0/20 (0)  CG: 8/20 (40)  P=0.003 | SG: 3/20 (15)  CG: 8/20 (40) | SG: 3/20 (15)  CG: 8/20 (40) |  |
| TRAUMA SURGERY | | |  |  |  |
| Ozmen | 1992 | NR | 7/43† (16) | 7/43† (16) | 4/14 patients had positive cultures upon aspiration from peritoneal cavity |
| GYNAECOLOGY | | |  |  |  |
| Waters | 2000 | 14/14 (100) | 13/14 (93) | 7/14 (50) |  |
| MIXED/OTHER | | |  |  |  |
| Jeng | 1998 | 8/8 | 8/8 | 8/8 (100) |  |

*Note.* BDA: Bile Duct Anastomosis, CG: Control Group, CVC: Central Venous Catheter, NR: Not Reported, SG: Study Group

* Only data extracted from Processed Red Cell Concentrate Portion 1 (PRBCC 1). PRBCC 2 is post-operatively collected.

† Units blood
