# Supplementary material for: Cell salvage in bacterially contaminated surgical fields – A scoping review
Source: PLoS One. 2026 Jan 5;21(1):e0339574. doi: 10.1371/journal.pone.0339574 (PMC12768276; doi:10.1371/journal.pone.0339574)
Supplement: S3 Table — (DOCX) [file pone.0339574.s003.docx]

**Supplemental Table 3**

*Quantification of bacterial load*

| First author | Year | Bacterial load before processing | | | Bacterial load after processing (final product). |  |
| --- | --- | --- | --- | --- | --- | --- |
|  | | |  | | | |
| Ezzedine | 1991 | NR | | | Positive cultures ≤2 CFU/mL: 42/51 (82%)  Positive cultures <5 CFU/mL: 48/51 (94%) |  |
| Schmidt^A^ | 2009 | CNS < 10^3/mL | | | CNS < 10^3/mL, Prop. a.e. |  |
|  | | |  | | | |
| Schmidt^A^ | 2009 | CNS a.e., MC < 10^2mL | | | CNS a.e., MC < 10^3mL |  |
|  | | |  | | | |
| Wollinsky | 1997 | Not specified | | | Mostly class 1 (broth only), one PRBCC1 with 130 CFU/mL (class 4) |  |
| Nosanchuk | 2001 | NR | | | 1 CFU/mL (17), 2 CFU/mL (1), 5 CFU/mL (1) |  |
|  | | |  | | | |
| Ozmen | 1992 | 9×10⁻⁵–5×10⁵ CFU/mL | | | 2×10⁻²–2×10⁴ CFU/mL |  |
|  | | |  | | | |
| Waters | 2000 | 3.0 [0.6–7.7]† | | | 0.1 [0.0–0.9]† |  |
| Teare | 2015 | 8 [1–84]§, total count 3.400 (1,278–52,200) | | | 3 [1–14]§, total 438 (98–2,115) |  |
|  | | |  |  | | |
| Locher | 1992 | NR | | | 1x10^5 3/18 (16,7%); 1x10^6 4/18 (22,2%); 1x10^7 10/18 (55.6%); >1x10^7 1/18 (5,6%) |  |
| Lenzen | 2006 | 2.8 × 10^4 ± 3.3 × 10^4 CFU/ml, with a maximum of 10^5 CFU/ml; 1.0 ± 0.6 species | | | No longer detected (detection limit 10^2 CFU/ml) |  |
|  | | |  | | | |
| Waters | 2003 | E. coli: 1,920 CFU/mL ± 452; S. aureus: 3,691 CFU/mL ± 5,152; P. aeruginosa: 1,970 CFU/mL ± 1,020; B. fragilis: 4,603 CFU/mL ± 1,480 | | | E. coli: 19 CFU/mL ± 16; S. aureus: 4 CFU/mL ± 7; P. aeruginosa: 0.6 CFU/mL ± 2; B. fragilis: 111 CFU/mL ± 74 |  |
| Yost | 2017 | ? | | | ? |  |
| Hinson | 2020 | E. coli = 1128 ± 634.52, S. pseudintermedius = 773.33 ± 210.09, P. aeruginosa = 1100.67 ± 529.51 | | | 0 CFU/mL (post-LRF2) |  |

*Note. a.e.: after enrichment,* CFU: Colony Forming Unit, CNS: Coagulase Negative Staphylococci, ICS: Intraoperative Cell Salvage, LRF: Leukocyte Reduction Filter, MC: Mixed Culture, NR: Not Reported, PRBCC: Packed Red Blood Cell Concentrate

†included for analysis

*units blood

‡from 20 different procedures from n=16 patients

§

^A^ studies discuss two different types of surgery and is therefore mentioned in two categories
