# Supplementary material for: Cell salvage in bacterially contaminated surgical fields – A scoping review
Source: PLoS One. 2026 Jan 5;21(1):e0339574. doi: 10.1371/journal.pone.0339574 (PMC12768276; doi:10.1371/journal.pone.0339574)
Supplement: S1 Appendix — (DOCX) [file pone.0339574.s005.docx]

# Appendices

**Appendix 1. Search strategies**

| **Database searched** | **Platform** | **Years of coverage** | **Records** | **Records after duplicates removed** |
| --- | --- | --- | --- | --- |
| Medline ALL | Ovid | 1946 - Present | 552 | 550 |
| Embase | Embase.com | 1971 - Present | 853 | 369 |
| Web of Science Core Collection* | Web of Knowledge | 1975 - Present | 539 | 88 |
| Cochrane Central Register of Controlled Trials | Wiley | 1992 - Present | 195 | 144 |
| **Total** | | | **2139** | **1151** |

*Science Citation Index Expanded (1975-present) ; Social Sciences Citation Index (1975-present) ; Arts & Humanities Citation Index (1975-present) ; Conference Proceedings Citation Index- Science (1990-present) ; Conference Proceedings Citation Index- Social Science & Humanities (1990-present) ; Emerging Sources Citation Index (2005-present)

No other database limits were used than those specified in the search strategies

**Medline**

(Operative Blood Salvage / OR blood salvage/ OR Blood Transfusion, Autologous / OR (((blood* OR erythrocyt*) ADJ3 (salvage* OR reinfus* OR re-infus* OR autolog*)) OR cell-save* OR cell-salvag* OR autotransfus* OR auto*-transfus*).ab,ti,kw.) AND (Equipment Contamination / OR * Infections/ OR Bacteremia/ OR Shock, Septic/ OR Bacterial Load/ OR ((bacter* ADJ3 (contamin* OR count* OR washout* OR wash-out* OR quantif*)) OR (infect* ADJ3 (risk* OR rate*)) OR bacterem* OR bacteraem* OR septic* OR sepsis*).ab,ti,kw. OR (contamin* OR bacter* OR infect*).ti.) NOT (news OR congres* OR abstract* OR book* OR chapter* OR dissertation abstract*).pt. NOT (exp * Stem Cells / OR exp * Stem Cell Transplantation / OR (stem-cell* OR precursor-cell* OR progenitor-cell*).ti.) AND english.la.

**Embase**

('intraoperative blood salvage device'/de OR 'blood salvage'/de OR 'blood autotransfusion'/de OR (((blood* OR erythrocyt*) NEAR/3 (salvage* OR reinfus* OR re-infus* OR autolog*)) OR cell-save* OR cell-salvag* OR autotransfus* OR auto*-transfus*):ab,ti,kw) AND ('bacterium contamination'/de OR 'medical device contamination'/de OR 'infection risk'/de OR 'infection rate'/de OR infection/mj OR bacteremia/exp OR 'septic shock'/de OR 'bacterial count'/de OR ((bacter* NEAR/3 (contamin* OR count* OR washout* OR wash-out* OR quantif*)) OR (infect* NEAR/3 (risk* OR rate*)) OR bacterem* OR bacteraem* OR septic* OR sepsis*):ab,ti,kw OR (contamin* OR bacter* OR infect*):ti) NOT ([conference abstract]/lim) NOT ('stem cell'/exp/mj OR 'stem cell transplantation'/exp/mj OR (stem-cell* OR precursor-cell* OR progenitor-cell*):ti) AND [english]/lim

**Web of science**

(TS=(((blood* OR erythrocyt*) NEAR/2 (salvage* OR reinfus* OR re-infus* OR autolog*)) OR cell-save* OR cell-salvag* OR autotransfus* OR auto*-transfus*)) AND (TS=((bacter* NEAR/2 (contamin* OR count* OR washout* OR wash-out* OR quantif*)) OR (infect* NEAR/2 (risk* OR rate*)) OR bacterem* OR bacteraem* OR septic* OR sepsis*) OR TI=(contamin* OR bacter* OR infect*)) NOT TI=((stem-cell* OR precursor-cell* OR progenitor-cell*)) NOT DT=(Meeting Abstract OR Meeting Summary) AND LA=(English)

**Cochrane**

((((blood* OR erythrocyt*) NEAR/3 (salvage* OR reinfus* OR re-infus* OR autolog*)) OR cell-save* OR cell-salvag* OR autotransfus* OR auto* NEXT transfus*):ab,ti,kw) AND (((bacter* NEAR/3 (contamin* OR count* OR washout* OR wash-out* OR quantif*)) OR (infect* NEAR/3 (risk* OR rate*)) OR bacterem* OR bacteraem* OR septic* OR sepsis*):ab,ti,kw OR (contamin* OR bacter* OR infect*):ti)

((stem-cell* OR precursor-cell* OR progenitor-cell*):ti)

("conference abstract":kw OR Trial registry record:pt)

#1 NOT #2 NOT #3
